# Two promoters in the *esx*-*3* gene cluster of *Mycobacterium smegmatis* respond inversely to different iron concentrations in vitro

**DOI:** 10.1186/s13104-017-2752-0

**Published:** 2017-08-25

**Authors:** Zhuo Fang, Mae Newton-Foot, Samantha Leigh Sampson, Nicolaas Claudius Gey van Pittius

**Affiliations:** 1 DST/NRF Centre of Excellence in Biomedical Tuberculosis Research, US/MRC Centre for Molecular and Cellular Biology, Division of Molecular Biology and Human Genetics, Department of Biomedical Sciences, Faculty of Medicine and Health Sciences, University of Stellenbosch, Francie van Zijl Drive, Tygerberg, 7505 South Africa; 2 Division of Medical Microbiology, Department of Pathology, Faculty of Medicine and Health Sciences, University of Stellenbosch, Tygerberg, South Africa; 3 National Health Laboratory Services, Tygerberg Hospital, Francie van Zijl Drive, Tygerberg, 7505 South Africa

**Keywords:** Iron, ESX, Tuberculosis, Mycosin-3, Promoter

## Abstract

**Background:**

The ESX secretion system, also known as the Type VII secretion system, is mostly found in mycobacteria and plays important roles in nutrient acquisition and host pathogenicity. One of the five ESXs, ESX-3, is associated with mycobactin-mediated iron acquisition. Although the functions of some of the membrane-associated components of the ESX systems have been described, the role of by mycosin-3 remains elusive. The *esx*-*3* gene cluster encoding ESX-3 in both *Mycobacterium tuberculosis* and *Mycobacterium smegmatis* has two promoters, suggesting the presence of two transcriptional units. Previous studies indicated that the two promoters only showed a difference in response under acid stress (pH 4.2). This study aimed to study the effect of a mycosin-3 deletion on the physiology of *M. smegmatis* and to assess the promoter activities in wildtype, mycosin-3 mutant and complementation strains.

**Results:**

The gene *mycP*_*3*_ was deleted from wildtype *M. smegmatis* via homologous recombination. The *mycP*_*3*_ gene was complemented in the deletion mutant using each of the two intrinsic promoters from the *M. smegmatis esx*-*3* gene cluster. The four strains were compared in term of bacterial growth and intracellular iron content. The two promoter activities were assessed under iron-rich, iron-deprived and iron-rescued conditions by assessing the *mycP*_*3*_ expression level. Although the *mycP*_*3*_ gene deletion did not significantly impact bacterial growth or intracellular iron levels in comparison to the wild-type and complemented strains, the two esx-3 promoters were shown to respond inversely to iron deprivation and iron rescue.

**Conclusion:**

This finding correlates with the previously published data that the first promoter upstream of *msmeg0615*, is upregulated under low iron levels but downregulated under high iron levels. In addition, the second promoter, upstream of *msmeg0620*, behaves in an inverse fashion to the first promoter implying that the genes downstream may have additional roles when the iron levels are high.

**Electronic supplementary material:**

The online version of this article (doi:10.1186/s13104-017-2752-0) contains supplementary material, which is available to authorized users.

## Background

Tuberculosis, whose etiological agent is *Mycobacterium tuberculosis* (*Mtb*), was one of the top ten causes of death worldwide in 2015 (1.4 million deaths) [1]. Such a tremendous medical burden is exacerbated by the emergence of multidrug-resistant TB (MDR-TB), thus new drug target is urgently needed for new anti-TB treatment development. An ideal drug target should be responsible for essential metabolic functions in *Mtb* and it should have no homology to human proteins to minimize drug toxicity to the host. The Type VII secretion systems, or ESXs (with five members ESX-1 to -5), are a signature group of protein secretion systems in mycobacteria. They have been extensively studied, especially ESX-1, -3 and -5, because they are responsible for bacterial survival and pathogenicity during *Mtb* infection [2]. Unlike ESX-1 and ESX-5, ESX-3 is most conserved in both pathogenic and environmental mycobacteria, and it is associated with mycobactin-mediated (an iron chelator secreted by the mycobacteria) iron acquisition [3–5], and also affects heme acquisition [6]. Abolishing both pathways would be a promising anti-tuberculosis therapeutic strategy [7].

**Fig. 1:**
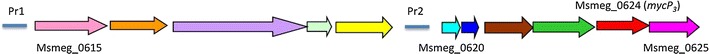
Genetic organization of the *esx*-*3* gene cluster in *Mycobacterium smegmatis*. The positions of the promoters, pr1 and pr2, are indicated(Adapted from [8])

The expression of the *esx*-*3* gene cluster in *M. tuberculosis* and *M. smegmatis* is governed by two promoters, the first located upstream of the first gene of the cluster (*msmeg_0615*) and the second located upstream of the *esx* genes (*msmeg_0620* and *msmeg_0621*) [8] (Fig. 1). The first promoter is controlled by the transcriptional regulator IdeR in an iron-dependent manner [9]. The regulator of the second promoter has not been identified. Previously, the activities of the two promoters in *M. smegmatis* were only shown to differ in response to acid stress (pH 4.2) and no difference was observed under iron-rich or iron-deprived conditions [8].

Compared to the other membrane protein components of the ESXs consisting of EccBCDE which constitute the core membrane structure [10], the roles played by mycosins remain elusive [2]. It was found that MycP_1_ cleaves EspB upon secretion, possibly facilitating the maturation of ESX substrates [11]. The stability of both the ESX-1 and ESX-5 complex could be compromised if MycP_1_ and MycP_5_ respectively, were absent, suggesting that mycosins are crucial for the integrity and functioning of the ESX [12]. However, how they facilitate the substrate secretion for their respective ESX systems remained poorly understood. The functional study on MycP_3_ is even more limited with no functional data published in the literature. In this report, *M. smegmatis* was used as a model organism in which *mycP*_*3*_ was deleted to generate the deletion mutant, and the *mycP*_*3*_ complementation strains were generated from the mutant by introducing *mycP*_*3*_ downstream of each of the two *esx*-*3* promoters. This study investigated the impact of the *mycP*_*3*_ deletion on bacterial growth and intracellular iron content under different iron conditions, as well as the activities of the two promoters under these conditions.

## Methods

### Bacterial strains, culture media and plasmid DNA

*Escherichia coli* XL-1 blue (Stratagene, USA, Catalogue No. 200249) was used for manipulating and propagating recombinant plasmid DNA. *Mycobacterium smegmatis* mc^2^ 155 (a gift from Rob Warren, South Africa) was used as the parent wildtype strain (WT_ms_) from which the MycP_3_ deletion mutant (ΔMycP_3ms_) was derived, and as template to generate two complementation strains (ΔMycP_3ms_::pr1MycP_3ms_ and ΔMycP_3ms_::pr2MycP_3ms_). *E. coli* was cultured using both Lysogeny Broth (LB) liquid media [1% (w/v) tryptone (Merck, USA, Catalogue No. 107213), 0.5% (w/v) yeast extract (Merck, USA, Catalog No. 113885), and 1% (w/v) sodium chloride (Sigma-Aldrich, USA, Catalogue No. S7653)] and solid media [LB liquid media supplemented with 1.5% (w/v) bacterial agar (Sigma-Aldrich, USA, Catalogue No. A5306)]. *M. smegmatis* was cultured using both Middlebrook 7H9 liquid medium (Becton–Dickinson, USA, Catalogue No. 221832) and Difco 7H11 solid medium (Becton–Dickinson, USA, Catalogue No. 212304) both supplemented with 0.05% (v/v) Tween 80 (Sigma Aldrich, USA, Catalogue No. P1754), 0.5% (w/v) glucose (Sigma Aldrich, USA, Catalogue No. 47829), and 0.5% (v/v) glycerol (Sigma-Aldrich, USA, Catalogue No. G5516). Fe-free 7H9 (omitting ferric ammonium citrate) and Fe-free Sauton’s medium (3.5 mM KH_2_PO_4_ (Sigma-Aldrich, USA, Catalogue No. NIST200B), 25 mM l-asparagine (Sigma-Aldrich, USA, Catalogue No. A0884), 10 mM citric acid (Sigma-Aldrich, USA, Catalogue No. 791725), 4 mM MgSO_4_·6H_2_O (Sigma-Aldrich, USA, Catalogue No. 746452), 5% (v/v) glycerol, and 0.05% (v/v) Tween-80) were also used to monitor bacterial growth under iron-limiting condition. The iron depleted 7H9 and Sauton’s media were prepared by mixing iron-free 7H9 and Sauton’s media (omitting MgSO_4_·6H_2_O) with 10 g/L Chelex resin (Bio-Rad, USA, Catalogue No. 1422822), a chelating agent, for 48 h and then filter-sterilized and supplemented with sterile MgSO_4_·6H_2_O (4 mM) before culturing *M. smegmatis*. Additionally, the iron deprivation rescuing of the *M. smegmatis* cultured in iron-deprived 7H9 or Sauton’s media was achieved by supplementation of ferric ammonium citrate in the same concentration of normal 7H9 or Sauton’s media.

The CloneJet1.2 vector (Thermofisher, USA, Catalogue No. K1231) was used for insert DNA amplification before cloning into the target vectors. The p2Nil suicide vector and the pGoal17 selection gene cassette [13] were used to generate ΔMycP_3ms_, and pMV306 [14] was used for MycP_3_ complementation (the three vectors were provided by Rob Warren as a gift).

### Construction of *M. smegmatis mycP*_*3*_ gene knockout strain and corresponding complemented strains

Homologous DNA recombination was used to generate the ΔMycP_3ms_ strain (unmarked in-frame deletion) as previously described [13]. One thousand basepair (bp) fragments upstream (UP) and downstream (DOWN) of *mycP*_*3ms*_ gene (MSMEG_0624) were amplified using Phusion^®^ DNA polymerase (ThermoFisher, USA, Catalogue No. F532S) with two pairs of primers (Table 1). The thermo-cycling conditions for producing these two PCR products were as follows: initial denaturation step at 95 °C for 30 s; 40 cycles of amplification at 95 °C for 5 s followed by 30 s at 60 °C and 1 min at 72 °C; final elongation step at 72 °C for 7 min. The UP and DOWN PCR fragments were blunt-end ligated into pJet1.2 vector individually according to the manufacturer’s instructions. The UP and DOWN DNA inserts were restriction digested out of pJet1.2 by *Hin*dIII/*Xho*I and *Xho*I/*Bam*HI restriction enzyme pairs respectively. The DNA inserts were simultaneously ligated into p2Nil, previously digested with *Hin*dIII and *Bam*HI, via three-way cloning (three pieces of DNA joining together) using T4 DNA ligase (Promega, USA, Catalogue No. M1801), resulting in recombinant p2Nil-UP-DOWN plasmid DNA. The selection gene cassette (P_Ag85_-*lac*Z P_hsp60_-*sac*B) from pGOAL17 was inserted at the *Pac*I restriction site p2Nil-UP-DOWN plasmid DNA and the final construct was electroporated into *M. smegmatis* mc^2^ 155 cells. Blue single-crossover colonies were selected on LB agar supplemented with 50 μg/mL kanamycin (Sigma-Aldrich, USA, Catalogue No. 17151) and 0.2% X-gal (Sigma-Aldrich, USA, Catalogue No. 11680293001). The colonies were picked and passaged in LB media in the absence of kanamycin to induce a second crossover event. Double crossover colonies were selected on LB agar supplemented with 5% sucrose (Sigma-Aldrich, USA, Catalogue No. E001888) and X-gal. White colonies were further screened by colony PCR using screening primers (Table 1) to distinguish between WT and ΔMycP_3ms_ strains. The colony PCR thermo-cycling conditions were as follows: initial denaturation step at 95 °C for 30 s; 40 cycles of amplification at 95 °C for 5 s followed by 30 s at 58 °C and 1 min at 72 °C; final elongation step at 72 °C for 7 min. The WT PCR product was approximately 1600 bp while that of the ΔMycP_3ms_ was about 200 bp (Additional file 1: Figure S1a).

**Table 1:** Primers used for ΔMycP_3ms_ generation, MycP_3_ complementation and RT-qPCR assay

Experiment	Gene related	Primer name	Primer sequence	Restriction site (underlined)
ΔMycP_3ms_ Generation	*mycP*_*3*_	UP forward	5′-AAGCTTTCCCACGCACATCG-3′	*Hin*dIII
		UP reverse	5′-CTCG AGATCACCTGTCGAGCACG-3′	*Xho*I
		DOWN forward	5′-CTCGAGATGACCGCCCGGATAGC-3	*Xho*I
		DOWN reverse	5′-GGATCCCCGGTCTCGGTGAC-3′	*Bam*HI
ΔMycP_3ms_ Construct verification	*mycP*_*3*_	*mycP*_*3*_ screening forward	5′-GCTCAACCCGAAGATC GCCTC-3′	N/A
		*mycP*_*3*_ screening reverse	5′-AGGAACATGCCTTTCCACCAGG-3′	N/A
MycP_3_ Complementa-tion	*mycP*_*3*_	pr1 forward	5′-CCATGGGACGCTGAACGAGTGTTTAC-3′	*Nco*I
		pr1 reverse	5′-GACGCCCAGACTCTTGTGGATCACATCGCGGTCGACCCGGGGCG-3′	N/A
		*mycP*_*3*_-pr1 forward	5′-CGCCCCGGGTCGACCGCGATGTGATCCACAAGAGTCTGGGCGTC-3′	N/A
		*mycP*_*3*_ reverse	5′-AAGCTTTCATGTGGTCTTGTCCTTCC-3′	*Hin*dIII
		pr2 forward	5′-CCATGGACGTGGGACGGCGACGA GAATC-3′	*Nco*I
		pr2 reverse	5′-GACGCCCAGACTCTTGTGGATCACGACTGTTTCC TTTCGAAGGTGGTG-3′	N/A
		*mycP*_*3*_-pr2 forward	5′-CACCACCTTCGAAAGGAAAC AGTCGTGATCCACAAGAGTCTGGGCGTC-3′	N/A
RT-qPCR assay	*sigA*	*sigA* forward	5′-GGGCGTGATGTCCATCTGCT-3′	N/A
		*sigA* reverse	5′-GTATCCCGGTGCATGGTC-3′	
	*mycP*_*3*_	*mycP*_*3*_ forward	5′-GGATCATCGCGTTCGTGGGTAC-3′	
		*mycP*_*3*_ reverse	5′-GTCTTGTCCTTCCGACGGTAGG-3′	
	*eccE*_*3*_	*eccE*_*3*_ forward	5′-GAGCCGTTGTTGACGGTTTG-3′	
		*eccE*_*3*_ reverse	5′-GTTCGGTCGACAACGGGTTC-3′	

The *M. smegmatis esx*-*3* gene cluster contains two promoters, namely pr1 and pr2 which are upstream of the MSMEG_0615 and the MSMEG_0620 genes, respectively (Fig. 1) [8]. Both promoters were used to make complementation constructs expressing MycP_3_ as previously described [15]. The PCR thermo-cycling conditions for making the pr1, pr2 and mycP_3_ respectively are as follows: initial denaturation step at 95 °C for 5 min, 40 cycles of amplification at 95 °C for 5 s followed by 30 s at 59 °C (pr1), 60 °C (pr2), and 62 °C (*mycP*_*3*_) and 1 min of elongation step at 72 °C; final elongation step at 72 °C for 7 min. The four pairs of primers for making the two complementation constructs in integrative pMV306 plasmid DNA are given in Table 1. The reverse primer sequences for amplifying the two promoters are partially complementary to the sense primers of *myc*P_3_ to facilitate single-joint PCR [16] connecting pr1/pr2 and MycP_3ms_. The thermo-cycling condition for single-joint PCR is as follows: initial denaturation step at 95 °C for 5 min, an annealing step at 55 °C for 15 min and the last elongation step at 72 °C for 3 min. The final joined PCR products were amplified using the following thermo-cycling condition: initial denaturation step at 95 °C for5 min, 40 cycles of 95 °C for 5 s followed by 62 °C for 30 s and elongation step at 72 °C for 2 min, and final elongation step at 72 °C for 7 min. The final PCR products, named pr1-mycP_3ms_ and pr2-mycP_3ms_ were ligated into the pMV306 vector respectively using T4 DNA ligase. The recombinant pMV306-pr1-*mycP*_*3*_ and pMV306-pr2-*mycP*_*3*_ plasmids were electroporated into the *M. smegmatis* ΔMycP_3ms_ mutant strain to generate two MycP_3_ complementation strains, ΔMycP_3ms_::pr1*myc*P_3ms_ and ΔMycP_3ms_::pr2*myc*P_3ms_. The genetic integrity of the WT, ΔMycP_3ms_ and two complementation strains were confirmed by colony PCR (See Additional file 1: Figure S1a). The thermo-cycling condition for the colony PCR was the same as that of the WT and KO strains except the annealing temperature was at 62 °C. The *eccE*_*3*_ gene is directly downstream of the *mycP*_*3*_ gene with a tetra-nucleotide overlap. The expression level of *eccE*_*3*_ gene was assessed via RT-qPCR to ensure there was no polar effect from *mycP*_*3*_ deletion (See Additional file 1: Figure S1b).

### Bacterial growth under iron-rich and iron-deprived conditions

*M. smegmatis* WT, ΔMycP_3ms_, ΔMycP_3ms_::pr1MycP_3ms_ and ΔMycP_3ms_::pr2MycP_3ms_ strains were cultured in 7H9 broth, iron-free 7H9 broth and Sauton’s media, iron-chelated 7H9 and Sauton’s media from a starting OD_600nm_ of 0.01. They were incubated at 37 °C with a rotating rate of 200 rpm for 48 h during which the OD_600nm_ reading was taken every 3 h. Complete iron depletion of the culture was reached by sub-culuring the bacteria three times in iron-free 7H9 or Sauton’s media and then finally into the iron chelated 7H9 or Sauton’s media. The growth curves were performed in biological triplicate.

### Intracellular iron quantitation

Intracellular iron quantitation was performed as previously described [17]. Fifty millilitres of bacterial cultures at mid-log phase (OD_600nm_ of 0.7–0.9) was harvested by centrifugation. The cell pellet was washed twice with cold Tris–HCl buffer [5 mM Tris (Sigma-Aldrich, USA, Catalogue No. T3253), pH 7.6, 0.005% (v/v) Tween 80] and then mixed with equal volume of 0.1 mm diameter glass beads (Biospec, USA, Catalogue No. 11079101) and resuspended in 500 μL 50 mM NaOH (Sigma-Aldrich, USA, Catalogue No. S8045). The mixture was ribolyzed using a FastPrep^®^-24 ribolyzer (MP Biomedicals, USA) at 6.0 m/s for 30 s. This was repeated three times with 30 s incubation on ice between each repeat. The whole cell lysate was cleared by centrifugation at 12,000×*g* at 4 °C for 30 min. One hundred microliters of the whole cell lysate was used for the iron quantification assay and another 25 μL was used for protein quantitation using the RC-DC protein assay (Bio-rad, USA, Catalogue No. 5000121). For iron quantitation, the whole cell lysate was transferred into one well of a 96-well microtiter plate and mixed with 100 μL of 10 mM HCl (Sigma-Aldrich, USA, Catalogue No. H3162) and then 100 μL iron-releasing reagent [a freshly made solution of equal volumes of 1.4 M HCl and 4.5%, KMnO_4_ (Sigma-Aldrich, USA, Catalogue No. 1.09121) in distilled water]. This mixture was incubated at 60 °C for 2 h, cooled to room temperature, and 30 μL of iron-detecting agent [6.5 mM ferrozine (Sigma-Aldrich, USA, Catalogue No. 160601), 6.5 mM neocuproine (Sigma-Aldrich, USA, Catalogue. N1501), 2.5 M ammonium acetate (Sigma-Aldrich, USA, Catalogue No. A1542), and 1 M ascorbic acid (Sigma-Aldrich, USA, Catalogue No. A7506)] was added to the well and incubated for 30 min at room temperature. The absorbance was read at 550 nm on a photospectrometer. Ferric chloride (Sigma-Aldrich, USA, Catalogue No. F2877) was used as iron standards at the concentration of 10–80 μM in 50 mM NaOH. The iron concentration was normalized against the protein content, which was done by dividing the iron concentration by the protein concentration, resulting in the unit of nmol (of iron) per mg (of protein). The experiment was performed in triplicate.

### RT-qPCR

Fifteen millilitres of each *M. smegmatis* strain was harvested at mid-log phase in normal 7H9, Fe-free 7H9 or Fe-rescued 7H9 by centrifugation. The supernatant was discarded and the pellet was resuspended in 1 mL FastRNA^®^ Blue solution (MP Biomedicals, USA, Catalogue No. 6025-050) and ribolyzed as described above. The whole cell lysate was cleared by centrifugation at 12,000×*g* at 4 °C for 30 min, and 700 μL of the supernatant was transferred into a new 1.5 mL tube and thoroughly mixed with 300 μL chloroform (Sigma-Aldrich, USA, Catalogue No. C7559). The mixture was centrifuged at 12,000×*g* at 4 °C for 10 min. The top aqueous layer was transferred to a new 1.5 mL tube and mixed with 500 μL pre-chilled 100% ethanol (Sigma-Aldrich, USA, Catalogue No. 900642). The mixture was transferred onto the RNA purification column from NucleoSpin^®^ RNA isolation kit (Macherey–Nagel, Germany, Catalogue No. 740955) and further total RNA purification was done according to manufacturer’s instructions. The quality of the total RNA was assayed using a Bioanalyzer (Agilent Technologies, USA) at the Central Analytical Facility (Stellenbosch University, South Africa).

Five micrograms of total RNA was treated with Turbo DNase (ThermoFisher, USA, Catalogue No. AM2238) according to the manufacturer’s instructions. One microgram of Turbo DNase-treated total RNA was used for cDNA synthesis using the PrimeScript™ 1st strand cDNA synthesis kit (TaKaRa, USA, Catalogue No. DRR037A) with the appropriate reverse primers (Table 1) as per manufacturer’s instructions. Quantitative PCR was conducted using SYBR^®^ *Premix Ex Taq*™ mastermix (TaKaRa, USA, Catalogue No. RR82WR) on a Bio-Rad CFX96™ Real-Time PCR Detection System (Bio-Rad, USA) with the following cycling conditions: initial denaturation at 95 °C for 30 s; 39 cycles of amplification at 95 °C for 5 s followed by 30 s at 60 °C. The subsequent melt curve started with a denaturation step at 95 °C for 10 s and then a melting step from 65 °C to 95 °C with 5 s staying at each 0.5 °C interval. *sigA* was selected as the reference gene due to its constitutive expression [18]. The expression of all genes of interest was normalized against that of *sigA* in the same RNA sample, which was done by dividing the number of cDNA copy number of *mycP*_*3*_ by that of *sigA*.

### ESX-3 promoter activity in response to iron levels

The promoter activity of the ESX-3 promoters in response to iron levels was assayed using RT-qPCR of the gene expression levels of *mycP*_*3*_ in WT, ΔMycP3_ms_, ΔMycP3_ms_::pr1MycP3_ms_ and ΔMycP3_ms_::pr2MycP3_ms_ strains under normal 7H9, iron deprived 7H9 and iron rescued 7H9 media. ΔMycP3_ms_ strain acted as the negative control.

### Statistical analysis

Differences of intracellular iron concentrations and gene expression levels of *mycP*_*3*_ between WT_ms_, ΔMycP3_ms_ and two complementation strains under different iron concentrations were evaluated by Two-way ANOVA using GraphPad Prism 5 software. The comparison was considered significant when *p* value is smaller than 0.05.

## Results

### *Mycobacterium smegmatis* ΔMycP_3_ mutant showed similar growth as the WT under low iron conditions

**Fig. 2:**
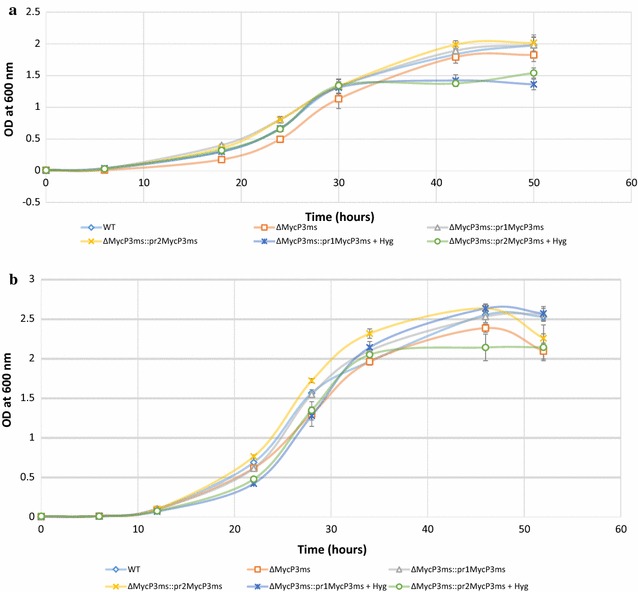
Growth curves of the WT, ΔMycP_3_ mutant and the two complementation strains, ΔMycP3_ms_::pr1MycP3_ms_ and ΔMycP3_ms_::pr2MycP3_ms_ under Fe-free 7H9 (**a**), Fe-free Sauton’s media (**b**). The growth curves were done in triplicate, *error bars* show standard deviation

**Fig. 3:**
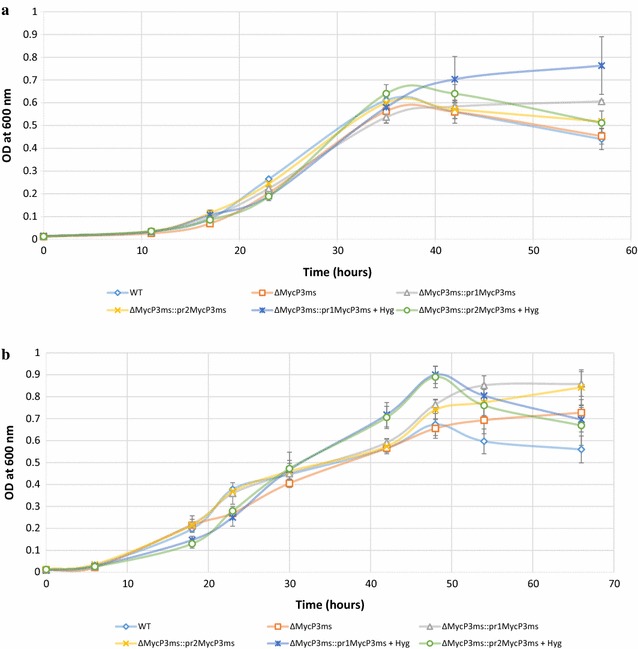
Growth curves of the WT, ΔMycP_3_ mutant and the two complementation strains, ΔMycP3_ms_::pr1MycP3_ms_ and ΔMycP3_ms_::pr2MycP3_ms_ under Fe-depleted 7H9 (**a**), and Fe-depleted Sauton’s media (**b**). The growth curves were done in triplicate, *error bars* show standard deviation

MycP_3_ is an important component of the ESX-3 protein secretion system although its detailed function has not been revealed. The growth profiles of *Mycobacterium smegmatis* WT, ΔMycP_3_ mutant and the two complementation strains, ΔMycP3_ms_::pr1MycP3_ms_ and ΔMycP3_ms_::pr2MycP3_ms_ were assessed under low iron and iron-deprived conditions in 7H9 and Sauton’s media to see whether the knockout of *mycP*_*3*_ gene would have a negative impact on the bacterial growth. However, no major difference in the exponential growth rate and the starting point of exponential growth phase was observed between the strains in these media, although ΔMycP_3_ mutant appears to have a slightly lower growth rate and OD_600_ reading at plateau than the WT and two complementation strains in these media except for iron-depleted Sauton’s medium (Figs. 2, 3). The differences of endpoint bacterial loads of all strains were observed, however, it was possibly due to bacterial clumping making the OD_600nm_ reading inaccurate. Clumping of all six cultures started to become visible when the growth reached plateau. It persisted although a range of Tween-80 concentrations and sonication intensity were applied to the culture (Results not shown).

### The *mycP*_*3*_ gene does not impact on bacterial intracellular iron level

**Fig. 4:**
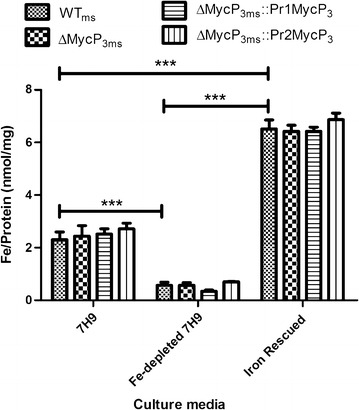
The comparison of the intracellular iron levels in WT_ms_, ΔMycP3_ms_, ΔMycP3_ms_::pr1MycP3_ms_ and ΔMycP3_ms_::pr2MycP3_ms_ strains under 7H9, Fe-depleted 7H9 and Fe rescued 7H9 media. The *error bars* show standard error of the mean (n = 4). The p values obtained using two-way ANOVA statistical analysis between different culturing conditions for all four strains are smaller than 0.0001 (***), an example is shown for the WT_ms_

Deletion of the *mycP*_*3*_ gene did not significantly affect the growth of ΔMycP_3ms_ under low iron conditions. But this does not rule out an impact on iron homeostasis, we therefore investigated whether *mycP*_*3*_ influenced bacterial iron acquisition by measuring intracellular iron levels. No significant differences between the strains were detected under three culturing conditions (Fig. 4). Intracellular iron levels dropped dramatically for all four strains after they were sub-cultured three times in Fe-free 7H9 and finally in Fe-depleted 7H9, showing an approximately 75% reduction. In contrast, intracellular iron level rose to a significantly higher level (approximately twofold) when iron was added to the Fe-depleted 7H9 media in the same concentration as conventional 7H9 medium.

### Functional analysis of the ESX-3 promoters

**Fig. 5:**
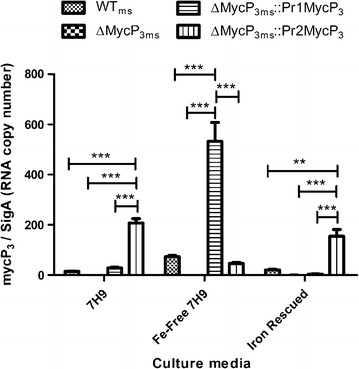
Gene expression analysis of *mycP*_*3*_ in WT_ms_, ΔMycP_3ms_, ΔMycP_3ms_::Pr1MycP_3_, and ΔMycP_3ms_::Pr2MycP_3_ strains under normal 7H9 (iron rich), Fe-free 7H9, and Fe-rescued 7H9 media. The results were normalized against the RNA copy number of *sigA*. The p values obtained using two-way ANOVA statistical analysis (n = 3) (*p < 0.05, **p < 0.01, ***p < 0.001, non-significant comparison is not shown)

We used both promoters from the *esx*-*3* gene cluster to construct the MycP_3_ complementation strains to see how the activities of the two promoters in the ESX-3 gene cluster in *M. smemgatis* differ in different iron levels. The promoters were incorporated into the two complementation strains separately and the strains did not show significant differences in either bacterial growth or mycobacterial intracellular iron levels under different iron concentrations (Fig. 4). We then assessed the promoter activity by determining the *mycP*_*3*_ gene expression levels in the four strains under different iron conditions (Fig. 5). In iron rich conditions, *mycP*_*3*_ under control of the first promoter was expressed at similar levels as observed in WT_ms_ while *mycP*_*3*_ expression is highly elevated under control of the second promoter. This expression profile was inverted in iron-deprived media, and restored when iron was added to the iron-deprived media (Fig. 5).

## Discussion

This study investigated the effect of the deletion of *mycP*_*3*_, an important component of the ESX-3 protein secretion system, on the physiology of *Mycobacterium smegmatis*. ESX-3 has been implicated in iron homeostasis via the mycobactin and heme iron acquisition systems as well as virulence through the secretion of the EsxG-EsxH and PE5-PPE4 protein pairs, and is essential for in vitro growth of *M. tuberculosis* [19, 20]. It could be a source of potential drug targets for anti-TB drug development.

The deletion of *mycP*_*3*_ alone did not affect the growth of *M. smegmatis* significantly or disrupt iron homeostasis, which correlates with the findings from Siegrist and colleagues [19]. However, deletion of ESX-3 makes the mycobacteria unable to grow under low iron conditions [4]. MycP_3_ possibly does not affect the secretion of the ESX-3 substrates significantly [19]. The deletion of *mycP*_*3*_ might influence mycobactin-mediated iron acquisition, but the iron acquisition overall was not disrupted because *M. smegmatis* possesses an alternative exochelin-mediated iron acquisition pathway. The exochelin biosynthesis and transporters are distinct from those of mycobactin [7] therefore exochelin-mediated iron acquisition may have compensated for any potential malfunction of mycobactin-mediated iron acquisition. Interestingly, a double mutant with *mycP*_*3*_ deleted and exochelin pathway disabled does not affect the bacterial growth in iron deprived media [19], suggesting that MycP_3_ is dispensable in the function of the ESX-3.

Surprisingly, the intracellular iron levels of all of the studied *M. smegmatis* strains, when iron rescued, were about twofold higher than when cultured in normal 7H9 media. This might be explained by the hypothesis that cell envelope-associated mycobactins serve as temporary storage for iron ions [21]. We reason that the production of mycobactin was suppressed under iron rich conditions through regulation by IdeR [9, 22] when first cultured in commercially available normal 7H9; but derepressed during iron deprivation to produce a large amount of mycobactin which was transported into the cell envelope. When iron was added to rescue the iron-starved bacterial culture, normal iron uptake was restored meanwhile the abundant cell envelope mycobactins were able to bind the available iron resulting in high cellular iron levels. In addition, the insignificant impact of the *mycP*_*3*_ gene knockout on the in vitro physiology of *M. smegmatis* is supported by the comparative proteomics between the WT_ms_ and ΔMycP_3ms_ under iron rich condition (Fang et al. unpublished results).

The *M. smegmatis* ESX-3 is expressed under the control of two promoters [8]. Previous studies did not find major differences in the activity of the promoters under various culturing conditions including iron-rich and iron-deprived, except during acid stress [8]. In this study, the two promoters responded to different iron levels in an inverse fashion. A possible reason for the discrepancy between our and Maciag’s data is possibly due to the different experiment setup as they used the expression of the gene directly downstream of the promoters (*msmeg0615* and *msmeg0620* respectively) as the reporters while we used *mycP*_*3*_ gene expression as the reporter and the promoter-mycP_3_ couple was independent of the *esx*-*3* cluster in the *M. smegmatis* genome. The transcription of *mycP*_*3*_ from the promoter (pr1) in the ΔMycP3_ms_::pr1MycP3_ms_ strain responds to different iron levels in the same fashion as WT_ms_ (Fig. 5) implying that the transcription of the *mycP*_*3*_ gene is controlled by the first promoter (pr1) even though the gene is downstream of the 2nd promoter (pr2) (Fig. 1). However, the transcription level of recombinant *mycP*_*3*_ under the control of the first promoter is significantly higher than endogenous *mycP*_*3*_ expression in WT_ms_. In the integrative complementation vector, the *myc*P_3_ gene was positioned directly downstream of the promoter, rather than being 9 genes downstream as in the WT_ms_ genome. Such an artificial genetic arrangement brings the gene closer to the transcription start site thereby increasing the rate of transcription, increasing the number of transcripts [23]. The second promoter is not regulated in the same manner as the first, suggesting its independence from IdeR regulation. It was proposed that the first promoter in the *esx*-*3* gene cluster is responsible for the transcription of the entire operon while the second promoter only influences the transcription of the 6 genes downstream of it [8]. Even when iron-deprived, the expression of *mycP*_*3*_ from the second promoter reached the same level as in WT_ms_. When iron was sufficient, the second promoter was up-regulated by an unknown mechanism, possibly to express the downstream ESX pair, MSMEG_0620 and MSMEG_0621, and secreted protein EspG_3_ (MSMEG_0622), which may be required in other metabolic pathways or even nutrient acquisition [24].

## Conclusion

Our study confirms that MycP_3_ is dispensable to the bacterial growth or iron homeostasis in *M. smegmatis* as previously shown in other studies. The two promoters in *esx*-*3* gene cluster respond inversely to iron-rich and iron-deprived conditions which was not observed previously implying that the two promoters are not redundant and the second promoter may regulate the production of the downstream genes for other metabolic activities in the bacteria which is of great interest for further investigation.

## Additional file

**Additional file 1: Figure S1.** (a) Colony PCR confirming the genetic integrity of the WT, ΔMycP_3ms_, ΔMycP3_ms_::pr1MycP3_ms_ and ΔMycP3_ms_::pr2MycP3_ms_ strains. ΔMycP_3ms_ screening primers (Table 1) were used to distinguish between WT and ΔMycP_3ms_, and the complementation strain generating primers were used to distinguish between ΔMycP3_ms_::pr1MycP3_ms_ and ΔMycP3_ms_::pr2MycP3_ms_ (pr1 forward and mycP_3_ reverse to screen for ΔMycP3_ms_::pr1MycP3_ms_, pr2 forward and mycP_3_ reverse to screen ΔMycP3_ms_::pr2MycP3_ms_) (Table 1). M: DNA Marker [1 kb DNA ladder Plus (Fermentas, USA)]; Lane 1: no template control; Lane 2 and 3: WT_ms_ (1674 bp); Lane 4 and 5: ΔMycP3_ms_ (251 bp); Lane 6 and 7: ΔMycP3_ms_::pr1MycP3_ms_ (1684 bp); Lane 8 and 9: ΔMycP3_ms_::pr2MycP3_ms_ (1509 bp). (b) Average negative log transformed ratio of copy number of transcripts of *eccE*_*3*_ gene and *sigA* gene in four strains cultured under normal 7H9 broth (n = 2).

## Abbreviations

WT: wildtype
ESX: ESAT-6 (6 kDa early secretory antigenic target) protein family secretion systems

## References

[1] WHO (2016). Global Tuberculosis Report 2016.

[2] Gröschel MI, Sayes F, Simeone R, Majlessi L, Brosch R (2016). ESX secretion systems: mycobacterial evolution to counter host immunity. Nat Rev Microbiol.

[3] Serafini A, Boldrin F, Palù G, Manganelli R (2009). Characterization of a Mycobacterium tuberculosis ESX-3 conditional mutant: essentiality and rescue by iron and zinc. J Bacteriol.

[4] Siegrist MS, Unnikrishnan M, McConnell MJ, Borowsky M, Cheng T-Y, Siddiqi N, Fortune SM, Moody DB, Rubin EJ (2009). Mycobacterial Esx-3 is required for mycobactin-mediated iron acquisition. Proc Natl Acad Sci USA.

[5] Tufariello JM, Chapman JR, Kerantzas CA, Wong K-W, Vilchèze C, Jones CM, Cole LE, Tinaztepe E, Thompson V, Fenyö D, Niederweis M, Ueberheide B, Philips JA, Jacobs WR (2016). Separable roles for *Mycobacterium tuberculosis* ESX-3 effectors in iron acquisition and virulence. Proc Natl Acad Sci USA.

[6] Serafini A, Pisu D, Palù G, Rodriguez GM, Manganelli R (2013). The ESX-3 secretion system is necessary for iron and zinc homeostasis in Mycobacterium tuberculosis. PLoS ONE.

[7] Fang Z, Sampson SL, Warren RM, Gey van Pittius NC, Newton-Foot M (2015). Iron acquisition strategies in mycobacteria. Tuberc Edinb Scotl..

[8] Maciag A, Piazza A, Riccardi G, Milano A (2009). Transcriptional analysis of ESAT-6 cluster 3 in *Mycobacterium smegmatis*. BMC Microbiol.

[9] Rodriguez GM, Voskuil MI, Gold B, Schoolnik GK, Smith I (2002). ideR, An essential gene in mycobacterium tuberculosis: role of IdeR in iron-dependent gene expression, iron metabolism, and oxidative stress response. Infect Immun.

[10] Houben ENG, Bestebroer J, Ummels R, Wilson L, Piersma SR, Jiménez CR, Ottenhoff THM, Luirink J, Bitter W (2012). Composition of the type VII secretion system membrane complex. Mol Microbiol.

[11] Ohol YM, Goetz DH, Chan K, Shiloh MU, Craik CS, Cox JS (2010). Mycobacterium tuberculosis MycP1 protease plays a dual role in regulation of ESX-1 secretion and virulence. Cell Host Microbe.

[12] van Winden VJC, Ummels R, Piersma SR, Jiménez CR, Korotkov KV, Bitter W, Houben ENG (2016). Mycosins are required for the stabilization of the ESX-1 and ESX-5 Type VII secretion membrane complexes. mBio 7.

[13] Parish T, Stoker NG (2000). Use of a flexible cassette method to generate a double unmarked *Mycobacterium tuberculosis* tlyA plcABC mutant by gene replacement. Microbiol Read Engl.

[14] Stover CK, de la Cruz VF, Fuerst TR, Burlein JE, Benson LA, Bennett LT, Bansal GP, Young JF, Lee MH, Hatfull GF (1991). New use of BCG for recombinant vaccines. Nature.

[15] Andreu N, Zelmer A, Fletcher T, Elkington PT, Ward TH, Ripoll J, Parish T, Bancroft GJ, Schaible U, Robertson BD, Wiles S (2010). Optimisation of bioluminescent reporters for use with mycobacteria. PLoS ONE.

[16] Yu J-H, Hamari Z, Han K-H, Seo J-A, Reyes-Domínguez Y, Scazzocchio C (2004). Double-joint PCR: a PCR-based molecular tool for gene manipulations in filamentous fungi. Fungal Genet Biol FG B.

[17] Riemer J, Hoepken HH, Czerwinska H, Robinson SR, Dringen R (2004). Colorimetric ferrozine-based assay for the quantitation of iron in cultured cells. Anal Biochem.

[18] Hu Y, Coates ARM (1999). Transcription of two sigma 70 homologue genes, sigA and sigB, in stationary-phase *Mycobacterium tuberculosis*. J Bacteriol.

[19] Siegrist MS, Steigedal M, Ahmad R, Mehra A, Dragset MS, Schuster BM, Philips JA, Carr SA, Rubin EJ (2014). Mycobacterial esx-3 requires multiple components for iron acquisition. mBio 5.

[20] Tinaztepe E, Wei J-R, Raynowska J, Portal-Celhay C, Thompson V, Philips JA (2016). Role of metal-dependent regulation of ESX-3 secretion in intracellular survival of *Mycobacterium tuberculosis*. Infect Immun.

[21] Ratledge C (2004). Iron, mycobacteria and tuberculosis. Tuberc Edinb Scotl.

[22] Gold B, Rodriguez GM, Marras SA, Pentecost M, Smith I (2001). The Mycobacterium tuberculosis IdeR is a dual functional regulator that controls transcription of genes involved in iron acquisition, iron storage and survival in macrophages. Mol Microbiol.

[23] Lim HN, Lee Y, Hussein R (2011). Fundamental relationship between operon organization and gene expression. Proc Natl Acad Sci USA.

[24] Ates LS, Ummels R, Commandeur S, van der Weerd R, Sparrius M, Weerdenburg E, Alber M, Kalscheuer R, Piersma SR, Abdallah AM, Abd El Ghany M, Abdel-Haleem AM, Pain A, Jiménez CR, Bitter W, Houben ENG (2015). Essential role of the ESX-5 secretion system in outer membrane permeability of pathogenic mycobacteria. PLoS Genet.

