# Supplementary material for: Two promoters in the esx-3 gene cluster of Mycobacterium smegmatis respond inversely to different iron concentrations in vitro
Source: BMC Res Notes. 2017 Aug 25;10:426. doi: 10.1186/s13104-017-2752-0 (PMC6389172; doi:10.1186/s13104-017-2752-0)
Supplement: Supplementary file 1 — Additional file 1: Figure S1. (a) Colony PCR confirming the genetic integrity of the WT, ΔMycP3ms, ΔMycP3ms::pr1MycP3ms and ΔMycP3ms::pr2MycP3ms strains. ΔMycP3ms screening primers (Table 1) were used to distinguish between WT and ΔMycP3ms, and the complementation strain generating primers were used to distinguish between ΔMycP3ms::pr1MycP3ms and ΔMycP3ms::pr2MycP3ms (pr1 forward and mycP3 reverse to screen for ΔMycP3ms::pr1MycP3ms, pr2 forward and mycP3 reverse to screen ΔMycP3ms::pr2MycP3ms) (Table 1). M: DNA Marker [1 kb DNA ladder Plus (Fermentas, USA)]; Lane 1: no template control; Lane 2 and 3: WTms (1674 bp); Lane 4 and 5: ΔMycP3ms (251 bp); Lane 6 and 7: ΔMycP3ms::pr1MycP3ms (1684 bp); Lane 8 and 9: ΔMycP3ms::pr2MycP3ms (1509 bp). (b) Average negative log transformed ratio of copy number of transcripts of eccE 3 gene and sigA gene in four strains cultured under normal 7H9 broth (n = 2). [file 13104_2017_2752_MOESM1_ESM.docx]

Additional files

2 kb

1.5 kb

1.2 kb

500 bp

300 bp

M

1

2

3

4

5

6

7

8

9


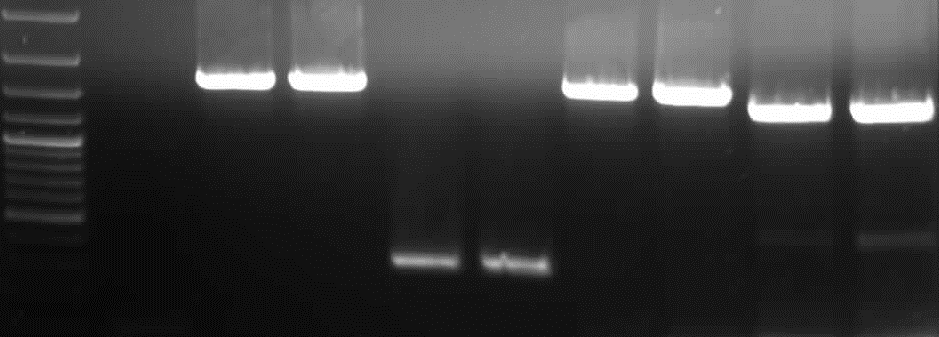


(a)

(b)

Figure S1. (a) Colony PCR confirming the genetic integrity of the WT, ΔMycP_3ms_, ΔMycP3_ms_::pr1MycP3_ms_ and ΔMycP3_ms_::pr2MycP3_ms_ strains. ΔMycP_3ms_ screening primers (Table 1) were used to distinguish between WT and ΔMycP_3ms_, and the complementation strain generating primers were used to distinguish between ΔMycP3_ms_::pr1MycP3_ms_ and ΔMycP3_ms_::pr2MycP3_ms_ (pr1 forward and mycP_3_ reverse to screen for ΔMycP3_ms_::pr1MycP3_ms_, pr2 forward and mycP_3_ reverse to screen ΔMycP3_ms_::pr2MycP3_ms_) (Table 1). M: DNA Marker [1 kb DNA ladder Plus (Fermentas, USA)]; Lane 1: no template control; Lane 2 and 3: WT_ms_ (1674 bp); Lane 4 and 5: ΔMycP3_ms_ (251 bp); Lane 6 and 7: ΔMycP3_ms_::pr1MycP3_ms_ (1684 bp); Lane 8 and 9: ΔMycP3_ms_::pr2MycP3_ms_ (1509 bp). (b) Average negative log transformed ratio of copy number of transcripts of *eccE_3_* gene and *sigA* gene in four strains cultured under normal 7H9 broth (n = 2).
